# Interaural speech asymmetry predicts bilateral speech intelligibility but not listening effort in adults with bilateral cochlear implants

**DOI:** 10.3389/fnins.2022.1038856

**Published:** 2022-12-07

**Authors:** Emily A. Burg, Tanvi D. Thakkar, Ruth Y. Litovsky

**Affiliations:** ^1^Waisman Center, University of Wisconsin-Madison, Madison, WI, United States; ^2^Department of Communication Sciences and Disorders, University of Wisconsin-Madison, Madison, WI, United States; ^3^Department of Psychology, University of Wisconsin-La Crosse, La Crosse, WI, United States; ^4^Division of Otolaryngology, Department of Surgery, University of Wisconsin-Madison, Madison, WI, United States

**Keywords:** listening effort, binaural hearing, pupillometry, speech intelligibility, bilateral cochlear implants, binaural redundancy, interaural asymmetry

## Abstract

**Introduction:**

Bilateral cochlear implants (BiCIs) can facilitate improved speech intelligibility in noise and sound localization abilities compared to a unilateral implant in individuals with bilateral severe to profound hearing loss. Still, many individuals with BiCIs do not benefit from binaural hearing to the same extent that normal hearing (NH) listeners do. For example, binaural redundancy, a speech intelligibility benefit derived from having access to duplicate copies of a signal, is highly variable among BiCI users. Additionally, patients with hearing loss commonly report elevated listening effort compared to NH listeners. There is some evidence to suggest that BiCIs may reduce listening effort compared to a unilateral CI, but the limited existing literature has not shown this consistently. Critically, no studies to date have investigated this question using pupillometry to quantify listening effort, where large pupil sizes indicate high effort and small pupil sizes indicate low effort. Thus, the present study aimed to build on existing literature by investigating the potential benefits of BiCIs for both speech intelligibility and listening effort.

**Methods:**

Twelve BiCI adults were tested in three listening conditions: Better Ear, Poorer Ear, and Bilateral. Stimuli were IEEE sentences presented from a loudspeaker at 0° azimuth in quiet. Participants were asked to repeat back the sentences, and responses were scored by an experimenter while changes in pupil dilation were measured.

**Results:**

On average, participants demonstrated similar speech intelligibility in the Better Ear and Bilateral conditions, and significantly worse speech intelligibility in the Poorer Ear condition. Despite similar speech intelligibility in the Better Ear and Bilateral conditions, pupil dilation was significantly larger in the Bilateral condition.

**Discussion:**

These results suggest that the BiCI users tested in this study did not demonstrate binaural redundancy in quiet. The large interaural speech asymmetries demonstrated by participants may have precluded them from obtaining binaural redundancy, as shown by the inverse relationship between the two variables. Further, participants did not obtain a release from effort when listening with two ears versus their better ear only. Instead, results indicate that bilateral listening elicited increased effort compared to better ear listening, which may be due to poor integration of asymmetric inputs.

## Introduction

Patients with cochlear implants (CIs) commonly report that listening is exhausting. This is because listening requires effort, defined as the intentional focus of cognitive resources to perform listening tasks (Pichora-Fuller et al., 2016). The amount of mental resources allocated can be influenced by many different variables, including the environment (e.g., quiet versus noisy) and individual factors such as linguistic skills, working memory capacity, and audibility (Wendt et al., 2016; Winn et al., 2018). Additionally, the amount of effort a listener expends is thought to be influenced by their motivation to perform the task (Pichora-Fuller et al., 2016; Hughes et al., 2018). Thus, two individuals listening to the same conversation may exert different amounts of effort depending on how motivated they are to pay attention and understand what is being said (Winn et al., 2018). Listening effort is an important aspect of communication to investigate because elevated effort is associated with fatigue and stress, especially for individuals who must overcome additional listening obstacles like hearing loss. Compared to individuals with normal hearing (NH), studies have found that individuals with hearing loss report higher levels of effort and fatigue, are more likely to require recovery after work, and are more inclined to take sick-leave due to stress-related factors (Kramer et al., 2006; Kramer, 2008; Nachtegaal et al., 2009; Alhanbali et al., 2017). Additionally, the subjective feeling that one needs to exert elevated effort in complex listening situations has been associated with feelings of social isolation and anxiety in individuals with hearing loss (Hughes et al., 2018).

Patients with severe-to-profound hearing loss who struggle to understand speech with a hearing aid can receive a cochlear implant (CI). An increasing number of patients with hearing loss in both ears are now being bilaterally implanted to maximize speech perception and improve spatial hearing abilities. Compared to hearing aids or a unilateral CI, most individuals with bilateral CIs (BiCIs) demonstrate improvements in sound localization (Gantz et al., 2002; van Hoesel and Tyler, 2003; Laszig et al., 2004; Litovsky et al., 2004, 2009; Nopp et al., 2004; Grantham et al., 2007; Tyler et al., 2007) and speech understanding in noise (Gantz et al., 2002; van Hoesel et al., 2002; van Hoesel and Tyler, 2003; Litovsky et al., 2004, 2009; Nopp et al., 2004; Schleich et al., 2004; Tyler et al., 2007; Loizou et al., 2009). Further, advantages of BiCIs have also been documented using subjective questionnaires. Tyler et al. (2009) administered the Spatial Hearing Questionnaire (SHQ) to bilateral and unilateral cochlear implantees and found that BiCIs users rated their localization, speech understanding in quiet, and music perception abilities significantly higher than unilateral CI users. Similarly, using the SHQ, Perreau et al. (2014) found that BiCI users reported better subjective hearing performance on individual spatial hearing items as well as sound localization, music, and speech understanding in quiet subscales compared to unilateral CI or bimodal CI users. Together, these findings suggest that bilateral implantation provides both objective and subjective benefit on a variety of listening tasks compared to unilateral implantation.

Binaural redundancy is another benefit that can be derived from having access to sound in both ears. This phenomenon arises from access to duplicate copies of a signal that can be combined centrally, resulting in improved speech intelligibility and an increase in perceptual loudness (Litovsky et al., 2006; Avan et al., 2015). Mosnier et al. (2009) found a binaural redundancy benefit of 10% in quiet for BiCI listeners using disyllabic word stimuli. Similarly, BiCI users in Laszig et al. (2004) demonstrated a binaural redundancy benefit of 4% using open-set sentence stimuli. In contrast, the same group of listeners in Laszig et al. (2004) did not show a significant binaural benefit using a different open-set sentence corpus, and BiCI users in Goupell et al. (2018) also did not demonstrate a binaural redundancy benefit using the IEEE sentence corpus. At least some of the variability in binaural redundancy benefit appears to be related to interaural asymmetry (either in speech intelligibility or hearing history). When Mosnier et al. (2009) split listeners into symmetric and asymmetric groups based on the difference in speech scores across ears, symmetric listeners (< 20% difference in percent correct across ears) demonstrated a significant binaural redundancy benefit, whereas asymmetric listeners did not. Yoon et al. (2011) used this same asymmetry criterion and measured binaural redundancy in quiet using sentences, consonants, and vowels. When averaging binaural redundancy for all three stimuli together, they observed significant benefit in quiet in symmetric BiCI users, but not asymmetric BiCI users. Likewise, listeners in Goupell et al. (2018) were recruited based on their asymmetric hearing history or early onset of deafness and late implantation. Together these results suggest that interaural asymmetry may preclude binaural redundancy benefits in quiet. However, due to methodological differences between studies (i.e., definition of “asymmetry,” stimuli used) this relationship warrants further investigation. We aim to examine this in the present study.

Historically, the primary measures of success regarding bilateral implantation have been bilateral speech intelligibility scores and spatial hearing abilities. There has been significantly less attention given to the potential impact of bilateral implantation on listening effort. Litovsky et al. (2006) administered a subjective questionnaire known as the Abbreviated Profile of Hearing Aid Benefit (APHAB) to BiCI users during a “bilateral deprivation” period in which participants only wore the CI of their better performing ear, and again several months later after participants had access to both of their CIs (Cox and Alexander, 1995; Litovsky et al., 2006). The APHAB contains 24 statements about everyday communication abilities or sound perception and asks participants to rate how often each statement is true. Statements are split into four subscales: Ease of Communication, Reverberation, Background Noise, and Aversiveness (Cox and Alexander, 1995). They found that participants perceived bilateral listening to be beneficial in background noise and reverberant environments and experienced increased ease of communication for bilateral compared to unilateral listening (Litovsky et al., 2006). Another study employed the Speech, Spatial, and Qualities of Hearing Scale, and found that individuals with two CIs expressed higher ability ratings on the spatial hearing domain, as well as segregation, naturalness, and listening effort aspects, compared to individuals with one CI (Noble et al., 2008). Together, these studies demonstrate that many patients subjectively experience reduced listening effort from BiCIs compared to a unilateral CI.

Another common method for quantifying listening effort is the behavioral dual-task paradigm. Hughes and Galvin (2013) used this method to assess listening effort during a speech-in-noise task in eight young BiCI users (aged 10–22 years) in unilateral and bilateral listening conditions. These listeners all had an early onset of deafness (before 1 year of age) and long inter-implant delays (mean = 7.8 years). They found that, on average, BiCI users demonstrated a significant reduction in listening effort when using two implants compared to one, however, on an individual level, this effect was only significant for three of the eight listeners (Hughes and Galvin, 2013). Another study asked 16 adult CI participants to repeat monosyllabic words in noise and found no difference in the dual-task or subjective measure of listening effort between unilateral CI and bimodal/bilateral CI listening (Sladen et al., 2018). Similarly, Perreau et al. (2017) found no difference in dual-task or subjective measures of listening effort between 10 unilateral CI users, 12 BiCI users, and 12 unilateral hybrid CI users. Due to the dearth of literature combined with the inconsistent results using either dual-task or subjective measures, we aimed to investigate listening effort with each CI alone and with BiCIs by measuring changes in pupil dilation. We chose this approach because pupillometry is considered to be an objective physiological measure of listening effort (Kramer et al., 1997; Zekveld et al., 2010; McGarrigle et al., 2014). To our knowledge, this is the first study to date that has examined this question using pupillometry.

Pupil dilation is modulated by cognitive load, increasing for difficult tasks that require more processing demand, and decreasing for tasks that are less challenging (Beatty, 1982). Mechanisms underlying the task-evoked pupil response include the activity of noradrenergic neurons in the locus coeruleus (Aston-Jones and Cohen, 2005). When the task becomes so difficult that listeners may feel that additional effort would not benefit performance, motivation declines, and pupil dilation decreases (Pichora-Fuller et al., 2016; Ohlenforst et al., 2017; Wendt et al., 2018). This effect has been shown for listening tasks that measure speech intelligibility. Pupil dilation increases as performance decreases to ∼30% correct, after which pupil dilation then decreases, presumably due to a decline in motivation and engagement (Ohlenforst et al., 2017; Wendt et al., 2018). Pupillometry is an ideal technique for studying listening effort in the hard of hearing population because it has the advantage of being compatible with assistive devices like hearing aids and CIs (Gilley et al., 2006; Friesen and Picton, 2010; Wagner et al., 2019). Additionally, unlike a dual-task paradigm, which is subject to behavioral bias and relies on a single metric such as response time (McGarrigle et al., 2014; Gagné et al., 2017), pupil dilation is completely objective and can be measured throughout the duration of a behavioral listening task to capture mental effort as it unfolds over time (Winn et al., 2018).

In short, the purpose of this study was to investigate the potential benefits of bilateral listening in performance and effort domains, both of which are important for successful communication. To do this, we measured speech intelligibility and listening effort in adults with BiCIs in three conditions: with their poorer ear only, better ear only, and bilaterally. Based on previous work that has shown binaural redundancy benefit in quiet (Laszig et al., 2004; Mosnier et al., 2009) and a reduction in listening effort for bilateral compared to unilateral CI listening (Litovsky et al., 2006; Noble et al., 2008; Hughes and Galvin, 2013), we predicted that speech intelligibility would be better (binaural redundancy) and pupil dilation would be smaller (release from effort) for BiCI users listening with both implants compared to their better ear only. Further, due to the accumulating evidence indicating an association between asymmetry and binaural benefits, we predicted that interaural speech asymmetry would be negatively related to binaural redundancy.

## Materials and methods

### Participants

Twelve native English-speaking adults with BiCIs were recruited to participate in this experiment (age range 25–78 years). Table 1 provides demographic information for these participants; 11 were implanted with Cochlear Ltd., devices, and one (IDI) was implanted with Advanced Bionics devices. Participants traveled to Madison, Wisconsin to participate in multiple studies over the course of several days. Testing for the present study took place over the course of one 2-h session. This study was approved by the University of Wisconsin-Madison Health Sciences Institutional Review Board.

**TABLE 1:** Participant demographics.

Subject ID	Sex	Age (years)	First implant	Better Ear	Inter-implant delay (years)	Bilateral CI experience (years)
ICW	F	25	Right	Right	18.6	4.9
IBZ	F	51	Right	Right	1.3	11.0
IDI	F	52	Right	Right	0.6	4.6
IBY	F	55	Left	Right	4.2	7.3
ICP	M	56	Left	Left	3	7.3
ICD	F	61	Right	Left	6.0	10.0
ICB	F	67	Right	Left	2.8	12.9
ICJ	F	69	N/A	Right	0.0	8.8
IDG	F	70	Left	Right	2.0	7.7
IBL	F	72	Left	Right	4.8	12.8
ICK	M	75	Right	Left	1.0	7.2
IBK	M	78	Left	Left	6.0	9.8

### Experimental setup

Testing took place in a standard sound booth (IAC Acoustics, IL, USA). Participants were seated at a table with their chin and forehead supported in a headrest to keep their head stable during testing; the table and chair position and height were adjusted for each participant. A computer monitor was attached to the table and positioned approximately 65 cm away from the headrest. The eyetracker camera was secured to the table using a desktop mount 8 cm in front of the monitor. Illumination of the test room was controlled for all participants (93 lux). Stimuli were played to a loudspeaker (Tannoy, Coatbridge, Scotland) positioned at 0° azimuth. Pupil size was measured in pixels using the “Area” setting on an eyetracker (Eyelink 1000 Plus; SR Research, Ontario, Canada) and a sampling rate of 1,000 Hz.

### Stimuli

Stimuli were drawn from the Institute of Electrical and Electronics Engineers sentence corpus (IEEE, 1969) and were recorded by a female talker. All stimuli were scaled to 65 dB SPL-A and played to the loudspeaker through a USB high-speed audio interface (RME Fireface, Haimhausen, Germany). Duration of sentences ranged from 4,000 to 6,000 ms. Custom software written in MATLAB (The MathWorks, Natick, MA, USA) with PsychToolbox 3 was used to deliver stimuli and collect data (Brainard, 1997; Kleiner et al., 2007).

### Procedure

Participants were tested in three listening conditions: better ear CI only (“Better Ear”), poorer ear CI only (“Poorer Ear”), and both CIs (“Bilateral”). Prior to testing, the better ear was classified as the ear with the higher word recognition score measured in the audiology clinic. If there was no difference in word recognition score between the two ears, the participant’s preferred ear according to subjective reporting was labeled the “better” ear. Participants were tested using their clinical programs with noise reduction and beamforming settings disabled. Before beginning the experiment, an informal interaural loudness balance check was completed with participants wearing both CI processors together to verify that they were equal in loudness. An experimenter stood directly in front of participants at the same distance as the loudspeaker and asked participants whether the ears were equally loud and sound was centered between the two ears. If participants perceived one CI to be noticeably louder than the other, the volume settings were adjusted so that the ears were balanced. Participants completed a familiarization procedure in which they listened to and repeated 10 sentences in each condition. Stimuli for practice trials were randomly selected and then excluded from the test corpus.

During testing, participants were asked to fixate their gaze on a small gray cross in the center of the computer screen and attend to open-set target sentences presented by a loudspeaker positioned directly in front of them (0° azimuth). Participants were instructed to repeat the sentence that was heard. Prior to the start of each trial, the gray cross turned white to indicate that the trial was about to commence. This was followed by a 2,000 ms pre-trial interval and then the trial began with a 1,000 ms baseline pupil measurement in silence before the stimulus (IEEE sentence) was presented. Following stimulus offset, participants were given a 2,000 ms silent period before the cross turned green and two beeps were presented, prompting participants to repeat what they heard. Each sentence contained five key words that were scored by an experimenter. The experimenter waited 10–15 s between trials to allow the pupil to return to baseline before beginning the next trial. Participants completed 30 trials per listening condition (30 sentences × 3 conditions = 90 sentences total). Trials were blocked into two runs per listening condition (15 sentences/run) and condition order was randomized for each participant. Target sentences were randomly selected from the corpus without replacement. Participants were given regular breaks during testing to avoid fatigue.

### Data analysis

Prior to data analysis, pupil data were pre-processed to reduce artifacts and discard noisy trials. First, pupil tracks with greater than 45% blinks were discarded from analysis (Burg et al., 2021). This blink criterion was chosen because it is more inclusive compared to other commonly used criteria (e.g., 15%, 30%). Previous work has shown a positive association between task difficulty and blink percentage; therefore, an overly conservative blink criterion like 15% could result in a higher number of difficult trials being excluded from analysis, potentially confounding results (Burg et al., 2021). When calculating the percentage of blinks in a track, samples from the response period were not considered since this part of the pupil track is influenced by the motor response (Privitera et al., 2010; Winn et al., 2015). Blinks were detected by tagging samples that fell below three standard deviations (SDs) from the mean (Zekveld et al., 2010). Consistent with best-practices described by Winn et al. (2018) tracks with irregular baselines, extreme distortions, or atypically large growth that is not consistent with task-evoked changes in pupil dilation were also discarded. In total, 1.4, 1.9, and 2.5% of trials were discarded due to these kinds of contamination for the Better Ear, Bilateral, and Poorer Ear condition, respectively.

The second step in pre-processing was an interpolation process, whereby individual tracks were “de-blinked” by linearly interpolating 80 ms before a blink and 160 ms following a blink to account for eyelid disturbances, and low-pass filtered using the “smooth” function in MATLAB (Zekveld et al., 2010). Next, raw pupil dilation was transformed to proportional change from baseline by subtracting the baseline value (average of first 1,000 ms of each trial) and then dividing by the baseline value. Baseline pupil dilation was compared across conditions to ensure that there were no systematic differences that would influence results. Divisive baseline correction was chosen over subtractive baseline correction because the former accounts for differences in pupil reactivity across participants and across trials for individuals (Winn et al., 2018). Finally, remaining tracks were time-aligned to stimulus offset and averaged together by listening condition for each participant. From the averaged trials, maximum pupil dilation and percentage of correctly repeated words were calculated and extracted for each condition. Maximum pupil dilation was extracted from the “silent period” (i.e., 2,000 ms period after stimulus offset and prior to response prompt), because this processing window has consistently been shown to elicit the largest pupil size during the trial for sentence recognition tasks (Zekveld et al., 2010; Winn et al., 2015, 2018).

### Statistical analysis

Speech intelligibility scores were transformed from percent correct to rationalized arcsine units (RAU) to alleviate ceiling effects and normalize variance (Studebaker, 1985). The effect of listening condition on speech intelligibility and listening effort were each evaluated separately using one-way repeated measure analysis of variance (ANOVA) tests with listening condition (three levels: Better Ear, Poorer Ear, Bilateral) as the independent variable. For these ANOVAs, dependent variables were either speech intelligibility (RAU) or maximum proportional change in pupil dilation (peak pupil size during the silent period). *Post hoc* pairwise comparisons were completed using paired *t*-tests. Benjamini-Hochberg corrections were employed to control false discovery rate (Benjamini and Hochberg, 1995). Correlational analyses were conducted to examine potential relationships between interaural speech asymmetry, change in speech intelligibility (RAU) from Better Ear to Bilateral conditions, and change in listening effort (pupil dilation) from Better Ear to Bilateral conditions. Assumptions for omnibus, *post hoc* tests, and correlations were statistically evaluated using Mauchly’s Test of Sphericity and Shapiro–Wilk normality tests. Due to our directional hypothesis that interaural speech asymmetry would be inversely related to change in speech intelligibility from the Better Ear to Bilateral condition (i.e., binaural redundancy), a one-sided test was used to evaluate this relationship. The relationship between interaural speech asymmetry and change in listening effort from Better Ear to Bilateral conditions was evaluated with a two-sided test. An alpha of 0.05 was used to determine whether results were statistically significant.

## Results

### Speech intelligibility

**FIGURE 1:**
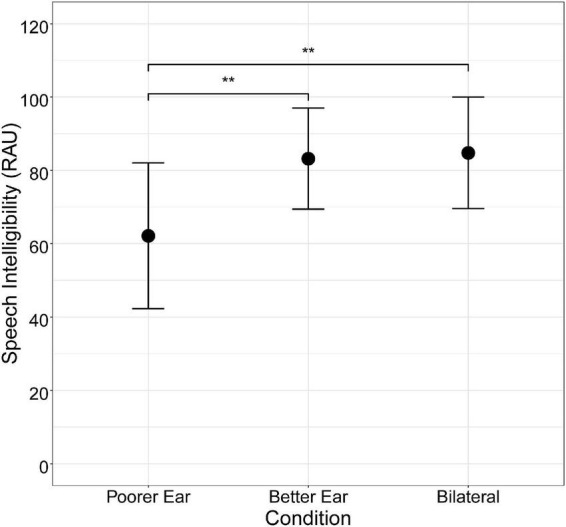
Mean speech intelligibility (RAU; *n* = 12) for each listening condition. Error bars represent ± 1.96 SE (95% confidence interval). Asterisks indicate the significance level of pairwise comparison results (* for *p* < 0.05, ** for *p* < 0.01, and *** for *p* < 0.001).

**FIGURE 2:**
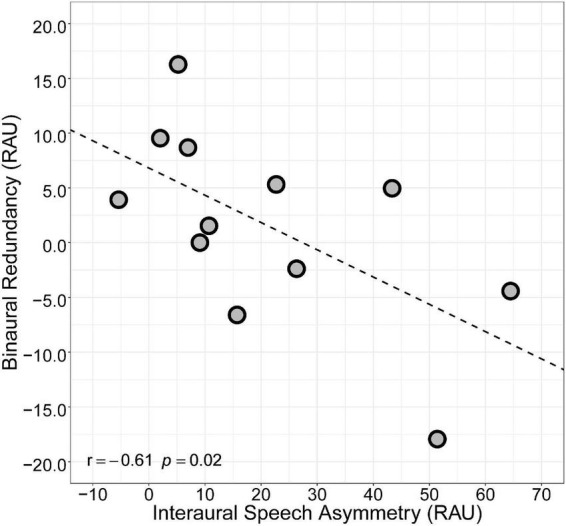
Relationship between interaural speech asymmetry (RAU) and binaural redundancy, defined as the difference in speech intelligibility (RAU) between Bilateral and Better Ear conditions.

Mean speech intelligibility (RAU) for each listening condition is shown in Figure 1. Speech intelligibility was higher for the Better Ear and Bilateral conditions than the Poorer Ear condition (Better Ear mean ± SD = 83.2 ± 24.4; Bilateral = 84.8 ± 26.9; Poorer Ear = 62.2 ± 35.2). Notably, there was substantial inter-subject variability in performance, as demonstrated by the wide range of performance (Better Ear = 21–105 RAUs; Bilateral = 19–114 RAUs; Poorer Ear = –5–104 RAUs) and large standard deviation for all conditions. There was also considerable variability in the amount of interaural asymmetry demonstrated by participants, which ranged from 2 to 65 RAUs (Table 2). A one-way repeated measures ANOVA using a Greenhouse–Geisser correction revealed a significant main effect of listening condition on speech intelligibility [*F*(2,22) = 13.4, *p* < 0.01]. *Post hoc* pairwise comparisons revealed that speech intelligibility did not significantly differ between Better Ear and Bilateral conditions (*p* = 0.55), indicating that, on average, participants did not demonstrate a binaural redundancy benefit. Further, speech intelligibility was significantly worse for the Poorer Ear condition compared to the Better Ear (*p* < 0.01) and Bilateral conditions (*p* < 0.01). Finally, Figure 2 plots binaural redundancy as a function of interaural speech asymmetry, with a higher positive value indicating greater binaural redundancy. Consistent with previous work reporting an association between asymmetry and binaural redundancy benefit (Litovsky et al., 2006; Mosnier et al., 2009; Yoon et al., 2011), a Pearson correlation revealed a significant negative relationship between the two variables, indicating that less speech asymmetry was associated with greater binaural redundancy benefit (*r* = –0.61, *p* < 0.05, one-tailed).

**TABLE 2:** Interaural speech asymmetry for each participant, defined as the difference in RAU scores between the Better Ear and Poorer Ear conditions.

	Subject ID											
	ICW	ICJ	IBL	ICP	ICK	ICB	IDG	IBK	IDI	ICD	IBY	IBZ
Interaural speech asymmetry	64.5	51.4	43.4	26.3	22.7	15.7	10.7	9.1	7.0	5.2	2.0	–5.4

### Listening effort

**FIGURE 3:**
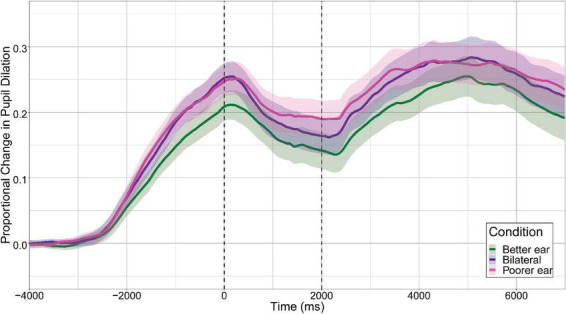
Grand average pupil tracks (*n* = 12) for each listening condition. Maximum proportional change in pupil dilation was extracted from the silent period, indicated by the vertical dashed lines (0–2,000 ms). Shaded regions represent ± 1.96 SE (95% confidence interval).

**FIGURE 4:**
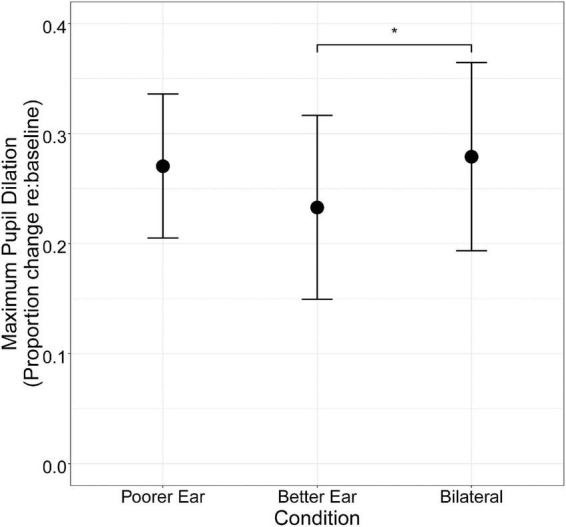
Mean maximum proportional change in pupil dilation (*n* = 12) for each listening condition. Error bars represent ± 1.96 SE (95% confidence interval). Asterisks indicate the significance level of pairwise comparison results (* for *p* < 0.05, ** for *p* < 0.01, and *** for *p* < 0.001).

**FIGURE 5:**
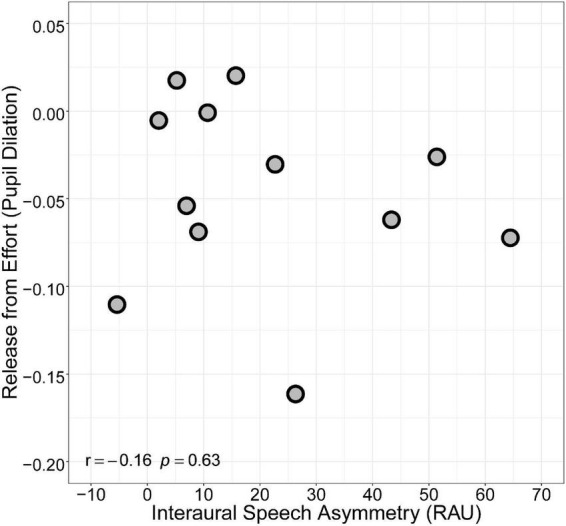
Relationship between interaural speech asymmetry (RAU) and release from listening effort, defined as the difference in maximum pupil dilation between Better Ear and Bilateral conditions.

Grand average pupil tracks for each condition (with 95% confidence intervals) are shown in Figure 3. In general, average pupil dilation during the silent period was largest for the Poorer Ear condition, followed by the Bilateral condition, and finally the Better Ear condition. Maximum pupil dilation was extracted from this period and is plotted in Figure 4. Maximum pupil dilation was smallest for the Better Ear condition and similar for Poorer Ear and Bilateral conditions (Better Ear = 0.23 ± 0.15; Poorer Ear = 0.27 ± 0.12; Bilateral = 0.28 ± 0.15). A one-way repeated measures ANOVA revealed that the main effect of listening condition was not significant [*F*(2,22) = 2.4, *p* = 0.1]. However, *F*-tests have the potential to lead to either false positives or false negatives; thus, the pairwise comparisons can be informative regardless of the omnibus result (Chen et al., 2018). Indeed, *post hoc* testing revealed that pupil dilation was significantly larger for the Bilateral condition compared to the Better Ear condition (*p* < 0.05). Contrary to our prediction, this indicates that participants exerted greater effort or engagement when listening bilaterally than with their better ear only. There were no significant differences between the Poorer Ear and Better Ear conditions (*p* = 0.24), or between the Poorer Ear and Bilateral conditions (*p* = 0.74). Finally, we examined whether interaural speech asymmetry was related to release from effort (Figure 5). Release from effort was calculated as the difference in maximum pupil dilation between the Better Ear and Bilateral conditions, with a higher positive value indicating a greater reduction in pupil dilation (and effort) when listening bilaterally. A Pearson correlation indicated that interaural speech asymmetry was not related to release from effort (*r* = –0.16, *p* = 0.63, two-tailed).

## Discussion

This study measured speech intelligibility and listening effort in adults with BiCIs to examine whether bilateral listening provides a benefit above the better ear alone. Speech intelligibility was significantly worse in the Poorer Ear condition compared to the Better Ear and Bilateral conditions, and there was no significant difference between performance in the Better Ear and Bilateral conditions. This indicates that, on average, the BiCI users in the present study had significant asymmetry in speech intelligibility across ears, but this asymmetry did not negatively affect performance in the Bilateral condition since performance was similar to the Better Ear condition. This is not surprising considering that listeners were tested in quiet and could rely on their better ear for speech intelligibility in the Bilateral condition. Further, pupil dilation was significantly larger in the Bilateral compared to Better Ear condition, and there was no significant difference between either of these and the Poorer Ear condition. This suggests that, on average, the BiCI users tested in this study did not obtain a performance benefit from binaural redundancy, nor did they obtain a release from effort when listening with two CIs versus their better ear alone.

### Interaural speech asymmetry predicts binaural redundancy benefit

The lack of measurable binaural redundancy benefit in the present study contrasts with results from Laszig et al. (2004) and Mosnier et al. (2009), which both reported significant binaural redundancy benefit for their BiCI listeners. However, there are noteworthy demographic differences between their participants and participants in the present study. Mosnier et al. (2009) required that their BiCI participants had less than a 5-year difference in duration of deafness between the two ears and were simultaneously implanted. Listeners in the present study, on the other hand, had variable differences in duration of deafness across ears, and inter-implant delays ranging from 0 to 18 years. Thus, our group of BiCI listeners was more heterogeneous and included listeners with asymmetric hearing histories. Similarly, participants in Laszig et al. (2004) did not demonstrate significant interaural speech asymmetry, whereas our BiCI participants exhibited large interaural speech asymmetries, with an average of 21.0 ± 22 RAU difference across ears. These observations indicate that interaural asymmetry may be key to understanding why our BiCI users, on average, did not demonstrate binaural redundancy. Consistent with this theory, Mosnier et al. (2009) and Yoon et al. (2011) split their participants into symmetric and asymmetric groups based on the difference in speech intelligibility across ears and found that only the symmetric groups demonstrated significant binaural redundancy benefit. Additionally, Goupell et al. (2018) failed to find a significant binaural redundancy benefit in BiCI listeners with asymmetric hearing histories or early onset of deafness and late implantation. These findings suggest that interaural asymmetries in hearing history and speech intelligibility may limit listeners’ ability to benefit from binaural redundancy. Indeed, we found that interaural speech asymmetry was inversely related to binaural redundancy in the present study (Figure 2), suggesting that the relatively large speech asymmetries demonstrated by our BiCI listeners (as compared to listeners in Laszig et al., 2004) may have limited their ability to successfully combine input from both ears and benefit from binaural redundancy.

**FIGURE 6:**
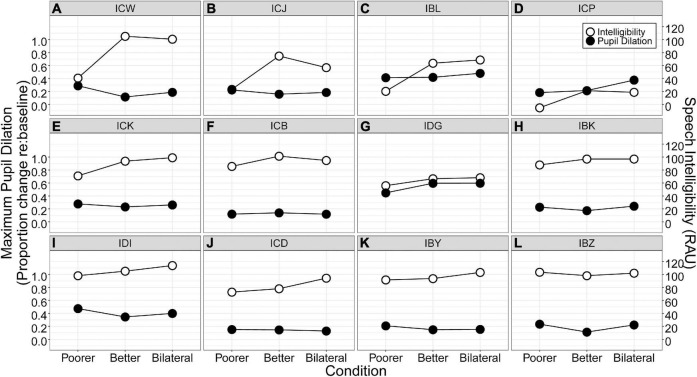
Speech intelligibility (RAU; open circles) and maximum proportional change in pupil dilation (closed circles) for each participant. Participants **(A–L)** are ordered from largest to smallest interaural speech asymmetry.

While binaural redundancy was not observed at the group level, the majority of listeners demonstrated improved performance in the Bilateral condition compared to the Better Ear condition. The largest binaural redundancy benefits were demonstrated by ICD (16 RAUs), IBY (10 RAUs), and IDI (9 RAUs; Figure 6). These listeners all demonstrated relatively small interaural asymmetries of 7 RAUs or less (Table 2). ICD had the second longest inter-implant delay of 6 years but also had 10 years of bilateral experience prior to testing, while IDI had the second shortest inter-implant delay of 0.6 years but only 5 years of bilateral experience prior to testing. In contrast, four listeners (ICW, ICJ, ICP, ICB) demonstrated worse performance in the Bilateral condition compared to the Better Ear condition. Three of these listeners demonstrated interaural asymmetries greater than 20 RAUs (Table 2), which was the percent correct criterion used by Mosnier et al. (2009) to categorize listeners into symmetric and asymmetric groups. The greatest decrement in performance from the Better Ear to Bilateral condition (18 RAUs) was shown by ICJ (Figure 6B). This participant had the second largest interaural asymmetry (51 RAUs). Interestingly, ICJ was simultaneously implanted, and had almost 9 years of bilateral CI experience prior to testing. In contrast, the participant with the largest interaural speech asymmetry (ICW: 65 RAUs; Figure 6A) and the longest inter-implant delay (18.6 years) only demonstrated a 4 RAU decrease in performance from the Better Ear to Bilateral condition. These are prime examples of the extreme variability that exists among BiCI users, and how difficult it can be to predict outcomes due to the vast number of variables that contribute to performance in each ear and across ears (Gantz et al., 2002; Litovsky et al., 2006; Mosnier et al., 2009).

### Bilateral listening is more effortful than better ear listening

Unlike previous studies that have shown that BiCIs may facilitate reduced listening effort compared to a unilateral CI (e.g., Litovsky et al., 2006; Noble et al., 2008; Hughes and Galvin, 2013), results from the present study indicate that, on average, bilateral listening elicited increased listening effort compared to better ear listening. In fact, out of the 12 BiCI participants tested, only two demonstrated a reduction in pupil dilation from the Better Ear to Bilateral condition (ICB and ICD, Figure 6, panels F and J). This is the first study to date that has shown this effect. Further, our results indicate that this increase in listening effort cannot be explained by a change in speech intelligibility, since there was no significant difference in performance between the Better Ear and Bilateral conditions. This is further supported by our correlation analysis that found no relationship between binaural redundancy and release from effort (*r* = 0.15, *p* = 0.65, two-tailed). Indeed, previous studies have shown differences in listening effort across conditions when speech intelligibility is held constant (e.g., Koelewijn et al., 2012). This underscores the value of measuring listening effort in studies examining speech intelligibility, as it can reveal additional information that is not apparent from performance alone.

To obtain a binaural redundancy benefit, listeners must be able to centrally combine information across ears (Litovsky et al., 2006). Results of the present study indicate that this ability was largely inaccessible to our group of BiCI users due to the large degree of interaural speech asymmetry observed. One reason that asymmetries may preclude binaural redundancy is that it is difficult to combine disparate signals into one coherent sound, which may in turn result in increased listening effort. In other words, increased effort in the Bilateral condition may be explained by a lack of binaural fusion. Steel et al. (2015) examined binaural fusion and listening effort in children with BiCIs. They found that poorer binaural fusion was associated with greater pupil dilation and longer reaction times. Further, larger brainstem asymmetries, classified by mismatched electrically evoked auditory brainstem latencies, were associated with worse binaural fusion abilities (Steel et al., 2015). Indeed, we also found a relationship between our measures of asymmetry (interaural difference in speech intelligibility) and binaural integration (binaural redundancy) (Figure 2). This suggests that increased listening effort in the Bilateral condition may be related to poor binaural fusion due to the relatively large interaural speech asymmetries demonstrated by our BiCI listeners. Pragmatically, it makes sense that attempting to integrate two disparate signals, or ignore an impoverished signal from the poorer ear, would require more effort than simply attending to the better ear alone. This theory is supported by previous work that has demonstrated impaired binaural fusion in BiCIs users (Fitzgerald et al., 2015) that is exacerbated by asymmetries, such as interaural place-of-stimulation mismatch (Kan et al., 2013). While degree of speech asymmetry was not significantly correlated with release from effort, this does not disqualify the possibility that the two are related in some way since the relationship was assessed using a simple linear correlation, and pupil dilation does not always scale linearly with task difficulty (Koelewijn et al., 2012; Ohlenforst et al., 2017; Wendt et al., 2018). Further, there is also evidence that BiCI users have abnormally broad pitch fusion ranges and that bimodal CI users with a hearing aid in the contralateral ear can experience interference, a decrease in performance when listening with two ears versus one. This may arise from involuntary fusion of disparate inputs (Reiss et al., 2016, 2018). Thus, it is also possible that BiCI users in the present study experienced unfavorable fusion, making it more difficult to understand the target speech. This effect might not have been reflected by speech intelligibility scores because listeners may have been able to compensate by using context clues to repair missing or ambiguous information, ultimately requiring more effort (Winn, 2016).

Alternatively, it is also possible that increased pupil dilation in the Bilateral condition represents increased engagement in the speech intelligibility task. Previous work has shown that pupil dilation increases with increasing task performance until the task becomes so difficult that increased effort is unlikely to improve performance (Ohlenforst et al., 2017; Wendt et al., 2018). In other words, listeners will continue to be engaged in a task so long as they perceive a potential benefit. Additionally, stimulus or task value to the participant can modulate engagement and pupil dilation even when speech is equally intelligible (Eckert et al., 2016; Winn et al., 2018). Because participants are accustomed to listening with both CIs in daily life, they may have expected to perform best in the Bilateral condition, resulting in increased pupil dilation due to greater engagement or motivation. Since we did not explicitly measure task engagement, we cannot disentangle engagement or motivation from effort in the present study. Another possibility is that increased loudness due to binaural summation contributed to greater pupil dilation in the Bilateral condition compared to either monaural condition. Indeed, Legris et al. (2022) demonstrated increasing maximum pupil diameter with increasing tone burst level (40 dBA, 60 dBA, 80 dBA) in both NH participants and hearing aid users. For NH participants, pupil dilation was significantly larger for all increases in level, but for hearing aid users, pupil dilation was only significantly different when comparing the 40 dBA condition to 80 dBA condition, regardless of whether or not participants were using their hearing aids. The 20 dB step size used by Legris et al. (2022) corresponds to a fourfold increase in loudness, whereas an increase of about 3 dB, as is typical for binaural summation, only corresponds to a 1.2-fold increase in loudness (Epstein and Florentine, 2012). Thus, the need to use large step sizes, especially in the hearing aid user group, indicates that the potential 3 dB of binaural summation experienced by BiCI users in the present study is very unlikely to have caused any significant change in pupil dilation. This is further supported by Nunnally et al. (1967) who only saw a significant effect of intensity on pupil dilation for very loud levels above 90 dB.

As mentioned previously, this is the first study to find bilateral listening to be more effortful than unilateral listening in BiCI users. One reason for the discrepancy between the present results and previous work may be the method used to gauge listening effort, as this was also the first study to investigate this question using pupillometry. In general, studies that have employed both subjective rating and pupillometry to measure effort have found that the two measures are typically uncorrelated (e.g., Zekveld et al., 2011; Zekveld and Kramer, 2014; Wendt et al., 2016). Lack of correspondence between these measures is likely related to participants’ subjective interpretation of what is “effortful” (Colby and McMurray, 2021). For example, some participants may base their effort rating on their performance accuracy rather than mental effort, resulting in a linear relationship between accuracy and subjective effort, whereas the relationship between accuracy and objective effort measured by pupil dilation has been shown to be non-monotonic (Koelewijn et al., 2012; Ohlenforst et al., 2017; Wendt et al., 2018; Winn and Teece, 2021). Finally, another important factor to consider is the unilateral comparison condition (i.e., Better versus Poorer Ear). The comparison between participants’ best possible unilateral listening condition (i.e., the Better Ear condition) and the Bilateral condition reveals changes explicitly due to bilateral listening. In contrast, if one were to compare the Poorer Ear condition to the Bilateral condition, it would be unclear whether changes in effort are simply due to the addition of the better ear or are explicitly related to bilateral listening. Litovsky et al. (2006) and Sladen et al. (2018) compared better ear listening to bilateral listening, but Hughes and Galvin (2013) did not report which ear their unilateral condition represented. If the unilateral condition represented the poorer performing ear or a mixture of poorer and better performing ears, a comparison of their results to the present study would be invalid.

## Limitations

The present study tested participants in quiet, which resulted in near-ceiling level performance for some listeners. While previous work has measured significant binaural redundancy benefit in BiCI users in quiet conditions (e.g., Laszig et al., 2004; Mosnier et al., 2009; Yoon et al., 2011) this benefit can be larger in noise conditions (e.g., Yoon et al., 2011). Measuring differences in speech intelligibility and pupil dilation from better ear to bilateral listening in both quiet and noise would elicit a wider range of performance and ultimately help elucidate whether bilateral CI listening is more effortful or more engaging than unilateral CI listening. Further, while subjective reports of listening effort do not always correlate with pupillometry results (e.g., Zekveld et al., 2011; Zekveld and Kramer, 2014; Wendt et al., 2016), it could nonetheless be interesting to compare the two metrics in future studies. Finally, a subjective measure that attempts to disentangle engagement/motivation from effort could be very useful for virtually any future study using pupillometry to gauge listening effort.

## Summary and conclusion

The present study measured speech intelligibility and pupil dilation to quantify differences in performance and listening effort in adults with BiCIs when listening with their poorer ear only, better ear only, or bilaterally in quiet. Previous studies have shown that some BiCI users demonstrate an increase in performance from better ear to bilateral listening. This was not observed in the present study, as BiCI users performed similarly when listening with their better ear only and bilaterally. The large interaural speech asymmetries demonstrated by our BiCI users may have precluded them from obtaining binaural redundancy benefit, as shown by the significant negative relationship between the two factors. Additionally, listeners exhibited an increase in pupil dilation for bilateral compared to better ear listening, indicating that bilateral listening was more effortful. Due to the substantial interaural asymmetries demonstrated by our participants (in speech intelligibility and hearing history) we propose that increased listening effort may be due to difficulty combining two disparate signals. In conclusion, these results indicate that interaural speech asymmetries can impede BiCI patients’ ability to access binaural redundancy and may provoke increased listening effort for bilateral compared to better ear listening. Therefore, investigating methods for reducing interaural asymmetries seems to be a promising direction for future research seeking to improve binaural hearing outcomes in BiCI patients.

## Data availability statement

The raw data supporting the conclusions of this article will be made available by the authors, without undue reservation.

## Ethics statement

The studies involving human participants were reviewed and approved by the University of Wisconsin-Madison Health Sciences Institutional Review Board. The patients/participants provided their written informed consent to participate in this study.

## Author contributions

EB, TT, and RL conceived the original idea and designed the study. RL supervised all aspects of the study. TT created custom software to collect and analyze the data. EB and TT tested the participants. EB processed the experimental data, performed the analysis, drafted the manuscript, and designed the figures with support and input from TT and RL. All authors discussed the results and provided critical feedback on the manuscript.
